# Notice to Our Readers

**Published:** 1895-08

**Authors:** 


### EDITORIAL.

### Notice to Our Readers.

Many of our readers failed to receive the July number of the Journal, but to those an explanatory letter was sent. It was solely because the Journal's exchequer could not afford to carry them any longer without reimbursement for their subscriptions. It will be the future policy of the business management of the Journal to exact a settlement of every subscription within the period of its existence.

